# Serum Levels and Glycosylation Changes of Alpha-1-Acid Glycoprotein According to Severity of Breast Cancer in Korean Women

**DOI:** 10.4014/jmb.2006.06007

**Published:** 2020-07-15

**Authors:** Jae Woong Choi, Ki-Ho Jeong, Ji Won You, Jun Woo Lee, Byung-In Moon, Hyoung Jin Kim, Hong-Jin Kim

**Affiliations:** 1 Laboratory of Virology, College of Pharmacy, Chung-Ang University, Seoul 06974, Republic of Korea; 2 Breast and Thyroid Cancer Center, Ewha Womans University Cancer Center for Women, Seoul 07985, Republic of Korea

**Keywords:** Alpha-1-acid glycoprotein, breast cancer, lectin, glycosylation

## Abstract

Elevated serum levels of alpha-1-acid glycoprotein (AGP) are known to be associated with several types of cancer. In addition, some reports have indicated that changes in glycosylation of AGP are associated with cancer progression. However, changes in AGP levels of serum and changes in glycosylation of AGPs in breast cancer have not been specifically studied. In the present study, serum AGP levels in benign (BN) cancer and breast cancer stage I (BC I), BC IIA, BC IIB, and BC III in Korean women were measured using an enzyme-linked immunosorbent assay (ELISA). AGP was purified from individual sera by hot phenol extraction and then subjected to AGP glycosylation analysis. Three types of AGP glycosylation (fucosylation, high-mannose-type and sialylation) were detected using enzyme-linked lectin assays (ELLAs). Serum AGP levels were higher in BC I, BC IIA, BC IIB, and BC III, than in the BN group, and the level in BC I and BC IIA was high enough to be distinguished from BN. Meanwhile, terminal fucosylation and high-mannose-type glycans appeared to be lowest in BC I. The glycosylation levels of BC I provide sensitivity and specificity that make BC I clearly distinguishable from BC IIA, BC IIB, and BC III as well as BN. Therefore, determination of serum AGP or AGP glycosylation level could be useful for detecting the early stages of breast cancer.

## Introduction

Human plasma alpha-1-acid glycoprotein (AGP, also known as orosomucoid) is an acidic (pKa = 2.6) glycoprotein that is highly soluble in water [1]. AGP is one of the most heavily glycosylated proteins in human plasma, and approximately 45% of its molecular weight (41–43 kDa) is composed of glycosylations [2]. It has been suggested to have anti-inflammatory or immunomodulatory activity, although its role in plasma is not clear [3]. Changes in the level of AGP in the blood are associated with systemic tissue injury, infection and inflammatory responses, and with an increase in hepatic AGP synthesis [4]. Therefore, it has been posited that expression of AGP affects mainly interleukin-1β (IL-1β), tumor necrosis factor-α (TNFα), interleukin-6 and IL-6-related cytokines [5, 6].

Serum AGP levels increase in various types of cancer. They are higher in hepatocellular carcinoma than in chronic liver disease [7], and are elevated in patients with gastric cancer compared to healthy volunteers [8]. There seems also to be an important link between ovarian cancer and elevated levels of AGP [9, 10] and it has been proposed that a decrease in AGP level is associated with remission of lung cancer, and an increase in AGP level with progression.

AGP has five N-glycosylation sites, at position 15, 38, 54, 75, and 85, respectively, with variable degrees of branching (mono-, bi-, tri-, and tetra-antennary glycans) [11]. Glycosylation of AGP is heterogeneous due to variable branch structures and multiple glycosylation sites [4]. Sialic acid, the most common carbohydrate, binds to the ends of the glycosylation branches of AGP and is linked to a galactose residue via either an α2-3 or α2-6 linkage [11]. Fucose is another terminating sugar found at the glycosylation end of AGP, and an increase in fucosylation is thought to cause an increase in four sialyl-Lewis epitope (LewisX) in acute and chronic inflammation [12].

There is evidence that changes in glycosylation of AGP are related to cancer progression. More highly-sialylated and fucosylated forms of AGP glycans are found in hepatocellular carcinoma and cirrhosis than in healthy controls, and levels of the glycoforms could be different enough to identify cancer stage [13]. Hashimoto *et al*. have suggested that malignancies with highly-fucosylated glycoforms of AGP for long periods after surgical resection have a poor prognosis while those with low levels of terminal fucosylation have a good prognosis [14]. Balmana *et al*. found that fucosylated glycoform levels are higher in serum AGP in pancreatic cancer than in healthy controls and chronic pancreatitis [15]. Shin *et al*. found more elevated levels of fucosylated glycans in cancer patients than in healthy controls, suggesting that the reduction in fucosylated forms might provide a way to evaluate the prognosis for surgery [16]. In summary, therefore, changes in the level of serum AGP, as well as changes in glycosylation of the AGP, are thought to be important indicators of cancer progression.

Breast cancer is the most common cancer in females; more than 2 million individuals worldwide are diagnosed with the disease every year, and it is the greatest cause of death in women [17]. In 2018, 627,000 women died of breast cancer, accounting for 15% of all female cancer deaths [17]. Moreover the incidence of breast cancer tends to be high in developed countries, and is increasing in almost all regions of the world [18]. Several blood-borne markers of breast cancer have been suggested. Examples are carcinoembryonic antigen (CEA), CA15-3 and cytokeratin fragments (TPA, TPS, and CYFRA 21-1), but none of these have sufficient specificity and sensitivity [19]. Major changes in branched glycosyl structures related to sialylation, fucosylation and mannosylation have been observed in the sera of breast cancer patients, indicating that changes in glycosylation are associated with metastasis and lymph node involvement [20].

The correlation between changes in serum AGP level and severity of cancer has not been properly studied in breast cancer although such a correlation has been proposed by studies in a number of cancers. In addition, changes in sialylation and fucosylation in blood are thought to be major events reflecting cancer progression. In this study, therefore, lectins were used to examine changes in AGP levels in serum as well as changes in levels of fucosylation and sialylation of AGP and of the high-mannose-type glycan as a function of the severity of breast cancer.

## Materials and Methods

### Ethics

This study was carried out with the approval of the Ewha Womans University Mokdong Hospital Institutional Review Board (Seoul, Korea, file No. 2019-01-027) and in accordance with the Declaration of Helsinki. Serum samples of patients were collected after obtaining written informed consents.

### Specimen Preparation

Sera from Korean women were collected at the Breast and Thyroid Cancer Center of Ewha Womans University Cancer Center for Women from November 2015 to January 2017. Serum samples were distributed as follows: benign breast disease (BN, *n* = 47) and breast cancer stage I (BC I, *n* = 48), IIA (BC IIA, *n* = 34), IIB (BC IIB, *n* = 12), and III (BC III, *n* = 25). Breast cancer stage was determined by the tumor-node-metastasis (TNM) staging system. Mean ages were BN group, 40, and BC I, IIA, IIB, and III, 51, 51, 55, and 54, respectively. The age range of the BN group was 19–74, and those of the BC I, IIA, IIB, and III groups were 29–78, 29–75, 33–83, and 36–78, respectively. Serum samples collected before surgical resection were centrifuged at 12,000 *g* for 10 min, and supernatants were collected and stored at −80°C.

### Extraction of AGP from Sera

AGP was extracted from sera by the hot phenol extraction method suggested by Chan and Yu [21]. Each serum sample (approximate volume: 20 μl) was combined with the same volume of 10 mM Tris-HCl pH 7.6 and mixed with 40 μl phenol solution saturated with distilled water. The mixtures were heated for 20 min at 70°C with shaking, chilled on ice for 10 min and centrifuged at 12,000 *g* for 10 min, and the supernatants were collected. The latter were mixed with the same volumes of stabilizing buffer (150 mM NaCl, 25 mM L-arginine) and stored at -80°C.

### Analysis of Purified AGP by SDS-PAGE

SDS-PAGE polyacrylamide gels were used to confirm the purity of the purified AGP, and the fractionated AGP was visualized by silver staining. The samples were diluted in phosphate-buffered saline (PBS) and subjected to 12.5% polyacrylamide gel electrophoresis with sodium dodecyl sulfate under reducing conditions. The molecular weights and purities of the various AGP fractions were assessed using commercial AGP from human plasma as standard (Sigma-Aldrich, USA, Cat. No. G9885).

### Serum AGP Measurement

Titers of AGP in serum samples were determined by indirect enzyme-linked immunoassay (ELISA). First, 96-well immunoplates (Greiner Bio-One, Germany) were coated with serial dilutions of serum in PBS at 37°C for 3 h, and the plates were blocked with 5% bovine serum albumin (BSA, MP Biomedicals, USA) in PBS containing 0.1% (v/v) Tween 20 (PBST) at 37°C for 2 h (300 μl for each well). Rabbit anti-AGP antibody (Abcam, ab118809, USA) diluted 1:5000 in 0.5% BSA in PBST was then added to the blocked plates at 37°C for 1.5 h. After washing, the plates were reacted with HRP-conjugated goat anti-rabbit IgG (Bethyl, A120-101P, USA). The secondary antibody was diluted 1:5000 in 0.5% BSA in PBST. The plates were washed three to five times with PBS-T between reactions. Color reactions were developed using *o*-phenylenediamine (Sigma-Aldrich; USA) and measured at 492 nm. End- point titers were set at an OD of 2 × the OD of the control well [22]. The control well consisted of blank without AGP sample.

### Titration of Purified AGP by ELISA

Titers of AGP were determined by the previous method with modifications [23]. Purified AGP was diluted with PBS from 1:200 to 1:51,200 and coated onto 96-well immunoplates (Greiner) at 37°C for 3 h. The coated wells were blocked with 5% BSA in PBST at 37°C for 2 h (300 μl for each well) and further treated as above. End-point titers were established at an OD of 2 × the OD of the control well [22]. The control well consisted of blank without AGP sample.

### Titration of Purified AGP by Enzyme-Linked Lectin Assay (ELLA)

Titers of fucosylated or sialylated AGPs or of high-mannose-type glycan were determined as previously, with modifications [23]. The samples were coated in the same manner as for ELISA and after washing with Tris- buffered saline (TBS) containing 0.1% Tween 20 (TBST), the wells were blocked with TBS containing 1% Tween 20 at room temperature for 2 h to avoid nonspecific reactions with lectins. Biotinylated lectins were purchased from Vector Laboratories (USA). Aleuria Aurantia Lectin (AAL) and jacalin were used to detect fucosylation and mannosylation, respectively, and Maackia Amurensis Lectin 2 (MAA) was used to detect sialylation. All lectins were diluted to 1 μg/ml in TBST and reacted with the AGP-coated wells at room temperature for 1 h, and bound lectins were detected with HRP-conjugated streptavidin (Thermo Fisher Scientific, 1:10,000 dilution). The plates were washed three to five times with TBST between reactions. Color reactions were developed as described above. End-point titers were set at an OD of 2 × the OD of the control well.

### Determination of Percentages of Lectin-Reactive AGPs

The percentages of lectin-reactive AGPs were calculated according to the formula: titer of AGP determined by ELLA (AAL-, jacalin- or MAA-reactive AGP) × 100/titer of AGP determined by ELISA. The titers of purified AGPs were used in these calculations. Data are presented as percentages.

### Statistical Analysis

Differences between groups were evaluated using Kruskal-Wallis tests, and *p* < 0.01 was considered significant. Area under the curve (AUC) values, *p*-value sensitivity, specificity, negative predictive value (NPV) and positive predictive value (PPV) were calculated using GraphPad prism version 5.01 (GraphPad Software, Inc., USA); cut- offs were selected to maximize the sum of sensitivity and specificity. Specificity = number of true negatives × 100/ number of true negatives + number of false positives. Sensitivity = number of true positives × 100/number of true positives + number of false negatives. Power was analyzed with the G Power 3.1 program (Franz Faul, Germany).

## Results

### Analysis of Serum AGP Levels in BN and BCs

The amounts of AGP in serum per group were determined by ELISA using the end-point titration method. The groups were classified as follows [24]: BN: unusual growths or other changes in breast tissue that do not contain cancer cells and with no invasion of surrounding tissues. BC I: invasive breast cancer where the cancer cells have started to penetrate normal surrounding breast tissue. In BC IIA, no tumor has been found in the breast, but cancers > 2 mm are found in 1–3 axillary lymph nodes, or tumors of 2 cm or less are found in the breast and the tumor has infiltrated the surrounding axillary lymph nodes, or tumors > 2 cm and < 5 cm are found in the breast, but no tumors have been found in the axillary lymph nodes. In BC IIB, tumors > 2 cm and < 5 cm are found in the breast and tumors > 0.2 mm and < 2 mm are found in the axillary lymph nodes, or tumors > 2 cm and < 5 cm are found in the breast and tumors are found in 1–3 axillary lymph nodes or around the breastbone, or tumors larger than 5 cm are found in the breast but no tumors are found in the axillary lymph nodes. In the case of BC III, tumors are found in 4 or more axillary lymph nodes or around breastbones, or tumors > 5 cm are found along with tumors in the axillary lymph nodes or around breastbones.

**Fig. 1:**
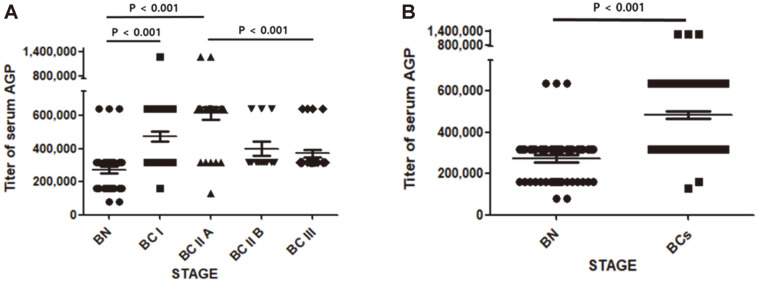
Titers of serum AGP. The titer of serum AGP in each group was determined by ELISA as described in Materials and Methods (**A**). Data are means ± standard error of the mean (SEM). BN, *n* = 47; BC I, *n* = 48; BC IIA, *n* = 34; BC IIB, *n* = 12; BC III, *n* = 25. *p* values < 0.01 were considered significant.

As shown in Fig. 1A, serum AGP titer showed a tendency to increase with increasing stage from BN to BC IIA, and then decrease. Overall, the AGP titer of the BC groups was higher than that of the BN group (Fig. 1B). Sensitivity, specificity, NPV, PPV, and AUC values for differentiating BC I, BC IIA, BC IIB, and BC III from BN are presented in Table 1. Sensitivity and specificity were higher, at 82% and 94%, respectively, when BC IIA was distinguished from BN, while sensitivities were 98, 100 and 100%, respectively, and specificity 40%, when BC I, BC IIB, and BC III were distinguished from BN. In addition, all of the AUC values when distinguishing the BC groups from BN had values ≥ 0.7. Thus it appears that measuring levels of serum AGP is effective as a way of detecting BCs.

### Purification of AGP from Serum Using Hot Phenol Extraction

**Fig. 2:**
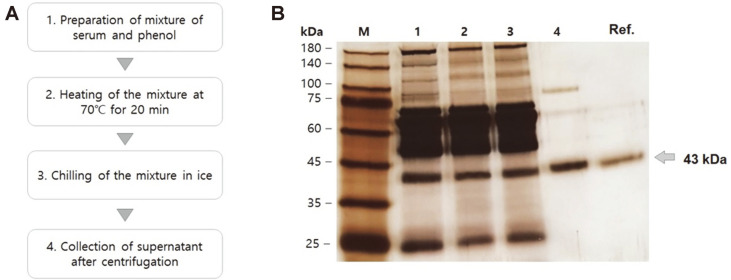
Scheme for purification of serum AGP. (**A**) The procedure for purification of AGP from serum by phenol extraction. (**B**) An SDS-PAGE analysis. M, molecular weight marker; 1, 2, 3, and 4 re samples after steps 1, 2, 3, and 4. Ref. refers to commercially available reference AGP.

AGP was extracted by the hot phenol extraction method [21] (see Fig. 2A). Serum was mixed with phenol and heated at 70°C for 20 min to precipitate contaminating proteins. After centrifugation, the precipitates were removed and pure AGP was obtained (Fig. 2A). Fig. 2B shows representative results of purification. The AGP was sufficiently pure to analyze its glycosylation (Fig. 2B).

### Analysis of Percentage of AAL-Reactive, Jacalin-Reactive or MAA-Reactive AGP

AGP has five N-glycosylation sites. In terms of lectin binding to N-glycosyls, AAL binds fucose attached to N- acetylglucosamine in α−1,6 linkage, while jacalin binds to high- mannose-type N-glycosyls [25]. MAA binds sialic acids attached to galactosyls in α−2,3 linkage [26]. Glycosylation of purified AGP was calculated as the percentage of AGP to which these lectins bound.

**Fig. 3:**
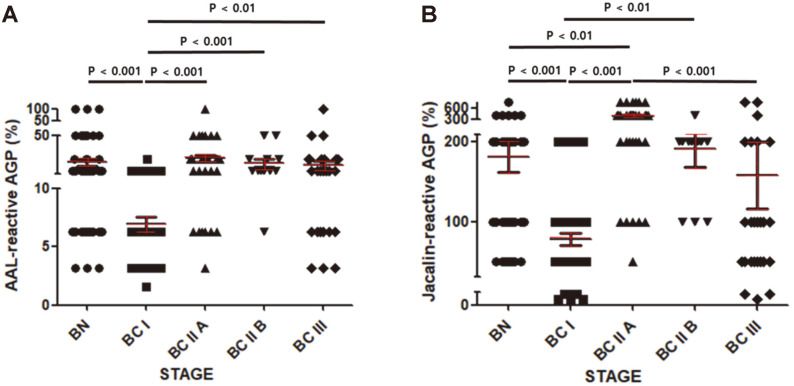
Percentages of AAL-reactive and jacalin-reactive AGP. Percentages of AAL-reactive AGP (**A**) and jacalin- reactive AGP (**B**) were determined as described in Materials and Methods. Data are mean ± SEM. BN, *n* = 47; BC I, *n* = 48; BC IIA, *n* = 34; BC IIB, *n* = 12; BC III, *n* = 25. *p* < 0.01 was considered significant.

**Fig. 4:**
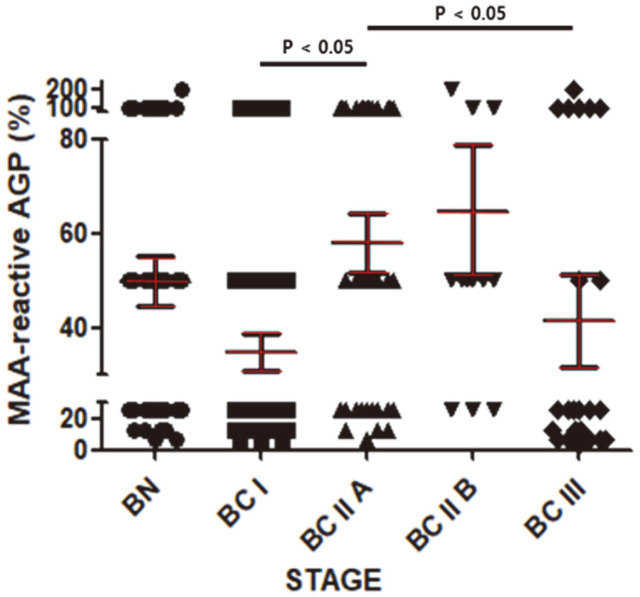
Percentages of sialylated AGP. Data are means ± SEM. Other details are as for Fig. 3.

As shown in Fig. 3A, the percentage of AAL-reactive AGP in the BC I group was significantly lower than in the BN group. This percentage increased significantly in the BC IIA group and was sustained from BC IIA to BC III (Fig. 3A). The trend was similar for the percentage of jacalin-reactive AGP but the values decreased from BC IIA to BC III (Fig. 3B). Even the percentage of MAA-reactive AGP in the BN I group tended to be lower than in the BN group (Fig. 4). Meanwhile, the percentage of MAA-reactive AGP showed a tendency to increase as the stage increased from BC I to BC IIA and then to BC IIB, finally decreasing in BC III (Fig. 4). The percentages of these lectin-reactive AGPs, and the statistical significance and power of the comparisons between groups are presented in Table 2.

A notable finding in Fig. 3 is that BC I can be discriminated from BN and from BC IIA, BC IIB, or BC III. This observation raises the possibility of detecting breast cancer at an early stage. Table 3 shows the sensitivity, specificity, NPV, PPV, and AUC values for distinguishing BCI from the other groups. Based on the percentage of AAL-reactive AGP in BC I versus the other groups, specificity ranged from 68 to 98% and sensitivity from 64 to 92%. Based on the percentage of jacalin-reactive AGP, specificity ranged from 60 to 88% and sensitivity from 56 to 82%. In both assays, an AUC value of 0.6 or higher was useful for distinguishing BC I from the other groups.

To see whether the percentages of lectin-reactive AGP and serum AGP titers calculated by ELISA for each group were affected by an age group bias, the correlation between these values and the age of each group was analyzed (Table 4). No clear correlation was found.

## Discussion

Neoplastic transformation is known to affect the structure of the various types of glycoprotein in cancer cells as well as the glycoproteins present in serum [27]. In the case of the latter, it is generally not easy to obtain individual glycosylation profiles because highly purified glycoprotein is required for the analysis of glycosylation [28], and this has been a barrier to the development of high-throughput assays.

Nevertheless, some attempts have been made to detect cancers through glycosylation analysis of unpurified serum glycoprotein. A representative example of the assay platforms used is the antibody-lectin sandwich assay used here that captures a target glycoprotein in serum using antibody-coated 96-well plates followed by detecting the glycosylation of the glycoprotein bound to the antibody with lectins [26]. In such antibody-lectin sandwich assays, glycosylation of the coating antibody and of glycoproteins present in the blocking agent (such as fetal bovine serum) can induce nonspecific signals. In our previous study of the glycosylation of serum CA15-3 using an antibody-lectin sandwich assay we found that only limited types of lectin had access to the captured CA15-3 [26]. The possibility that glycan structures present in unpurified glycoprotein are hidden or masked has been suggested as an explanation for their reactivity with only limited types of lectins [29].

In this study, we tried to analyze glycosylation of glycoprotein according to the severity of breast cancer by purifying glycoprotein from the sera of Korean women with BN and BCs. As glycoprotein had to be purified from the blood of 166 individuals, it needed to be present at a sufficient level in blood, and purification also had to be easy. AGP has a blood concentration of 50–100 mg per liter and can be efficiently purified from the blood by phenol extraction [30, 31], so this assay met the above-mentioned demanding conditions.

Our results suggest that distinguishing BCs from BN is possible using the increase in AGP level in the blood as an indicator (Table 1). In addition, it appeared that the reactivity to AAL and jacalin of purified BC I AGP was significantly lower than that of BN, BC IIA, BC IIB or BC III (Fig. 3), indicating that the BC I stage is associated with an important glycosylation change in AGP. Previous studies have indicated that glycosylation changes in AGP have a significant effect on its physiological functions [7, 32]. In addition, the immunomodulatory properties of AGP appear to be dependent on its glycosylation: extensive branching of glycans on AGP inhibits the proliferation of lymphocytes, and also induces an inhibitor of IL-1 of macrophages [33-35].

BC I is defined as the stage at which cancer cells begin to invade surrounding normal tissue. In our results, the terminal sialylation, fucosylation, and mannosylation of AGP glycans appeared to decline in BC I (Figs. 3 and 4). Meanwhile, the level of AGP in the blood increased from BC I (Fig. 1). The combination of these two changes could thus make AGP an effective blood marker for BC I. Moreover, AGP may be part of a surveillance system for cancer because it is involved in immunomodulation. In the future, if the reasons for the increased expression of AGP with BC grade and for the change in glycosylation become better understood, early detection of BC could be greatly improved.

The 5-year survival rate in BC I is 100% and for BC II it is 93%. Therefore, the discovery of markers capable of detecting BC early is important for maintaining the survival rate of BC. According to the results in Fig. 1, serum AGP increased up to BC IIA and then decreased in BC IIB and BC III. The features distinguishing BC IIA from BC IIB are an increase in tumor size and an increase in the size of tumors in the axillary lymph nodes. In a previous study, attention was drawn to the immunomodulatory action of AGP. From this point of view, it is thought that the decrease in serum AGP level with increasing BC stage is related to the failure of immune modulation (Fig. 1A). Overall, the combination of AGP level in blood and the changes in glycosylation of AGP promise to facilitate early detection of BC1.

## Figures and Tables

**Table 1:** Sensitivity, specificity, NPV and PPV for discriminating BCs from BN based on serum AGP titration.

Comparison	Sensitivity	Specificity	NPV	PPV	AUC value
BN vs. BC I	98%	40%	95%	62%	0.8
BN vs. BC II A	82%	94%	88%	90%	0.9
BN vs. BC II B	100%	40%	100%	30%	0.7
BN vs. BC III	100%	40%	47%	35%	0.7
BN vs, BCs	48%	94%	85%	42%	0.8

**Table 2:** Statistical parameters for comparison of each group.

Type of assay	Graph	Compared groups	Significance	Power value
Serum AGP titer	Fig. 1A	BN vs. BC I	*p* < 0.001	1.0
		BN vs. BC II A	*p* < 0.001	1.0
		BN vs. BC II B	N/A	N/A
		BN vs. BC III	N/A	N/A
		BC I vs. BC II A	N/A	N/A
		BC I vs. BC II B	N/A	N/A
		BC I vs. BC III	N/A	N/A
		BC II A vs. BC II B	N/A	N/A
		BC II A vs. BC III	*p* < 0.001	1.0
		BC II B vs. BC III	N/A	N/A
% of AAL-reactive AGP	Fig. 3A	BN vs. BC I	*p* < 0.001	1.0
		BN vs. BC II A	N/A	N/A
		BN vs. BC II B	N/A	N/A
		BN vs. BC III	N/A	N/A
		BC I vs. BC II A	*p* < 0.001	1.0
		BC I vs. BC II B	*p* < 0.001	1.0
		BC I vs. BC III	*p* < 0.01	0.9
		BC II A vs. BC II B	N/A	N/A
		BC II A vs. BC III	N/A	N/A
		BC II B vs. BC III	N/A	N/A
% of jacalin-reactive AGP	Fig. 3B	BN vs. BC I	*p* < 0.001	1.0
		BN vs. BC II A	*p* < 0.01	1.0
		BN vs. BC II B	N/A	N/A
		BN vs. BC III	N/A	N/A
		BC I vs. BC II A	*p* < 0.001	1.0
		BC I vs. BC II B	*p* < 0.01	1.0
		BC I vs. BC III	N/A	N/A
		BC II A vs. BC II B	N/A	N/A
		BC II A vs. BC III	*p* < 0.001	1.0
		BC II B vs. BC III	N/A	N/A
%s of MAA-reactive AGP	Fig. 4	BN vs. BC I	N/A	N/A
		BN vs. BC II A	N/A	N/A
		BN vs. BC II B	N/A	N/A
		BN vs. BC III	N/A	N/A
		BC I vs. BC II A	*p* < 0.05	0.9
		BC I vs. BC II B	N/A	N/A
		BC I vs. BC III	N/A	N/A
		BC II A vs. BC II B	N/A	N/A
		BC II A vs. BC III	*p* < 0.05	0.3
		BC II B vs. BC III	N/A	N/A

Comparative sets statistically significant were presented as red letters.

**Table 3:** Sensitivity, specificity, NPV and PPV for discriminating BC I from BN, BC IIA, BC IIB, and BC III using percentage of AAL- or jacalin-reactive AGP.

Type of assay	Comparison	Sensitivity	Specificity	PPV	NPV	AUC
% of AAL- reactive AGP	BC I vs. BN	77%	68%	71%	74%	0.8
	BC I vs. BC II A	65%	98%	96%	80%	0.9
	BC I vs. BC II B	92%	77%	50%	97%	0.9
	BC I vs. BC III	64%	77%	59%	80%	0.8
% of jacalin- reactive AGP	BC I vs. BN	60%	85%	81%	68%	0.8
	BC I vs. BC II A	82%	88%	82%	88%	0.9
	BC I vs. BC II B	75%	88%	60%	93%	0.9
	BC I vs. BC III	56%	60%	42%	73%	0.6

**Table 4:** Correlation of age and values of each assay.

		Benign (*n* = 47)	Stage I (*n* = 48)	Stage IIA (*n* = 34)	Stage IIB (*n* = 12)	Stage III (*n* = 25)
	Range of age	19-74	29-78	29-75	33-83	36-78
	Mean age	40	51	51	55	54
Pearson, r	Serum AGP titer	-0.19	0.00	-0.09	0.00	0.09
	% of AAL-reactive AGP	0.20	-0.11	-0.13	-0.3	-0.24
	% of jacalin-reactive AGP	0.17	-0.1669	-0.16	-0.09	0.28
	% of MAA-reactive AGP	0.26	-0.1	-0.15	-0.18	-0.16

## References

[1] Heegaard PM, Miller I, Sorensen NS, Soerensen KE, Skovgaard K (2013). Pig alpha1-acid glycoprotein: characterization and first description in any species as a negative acute phase protein. PLoS One.

[2] Schmid K, Nimerg RB, Kimura A, Yamaguchi H, Binette JP (1977). The carbohydrate units of human plasma alpha1-acid glycoprotein. Biochim. Biophys. Acta.

[3] Hochepied T, Berger FG, Baumann H, Libert C (2003). Alpha(1)-acid glycoprotein: an acute phase protein with inflammatory and immunomodulating properties. Cytokine Growth Factor Rev..

[4] Bteich M (2019). An overview of albumin and alpha-1-acid glycoprotein main characteristics: highlighting the roles of amino acids in binding kinetics and molecular interactions. Heliyon.

[5] Kwon H, Pessin JE (2013). Adipokines mediate inflammation and insulin resistance. Front. Endocrinol. (Lausanne).

[6] Lumeng CN, Saltiel AR (2011). Inflammatory links between obesity and metabolic disease. J. Clin. Invest..

[7] Bachtiar I, Santoso JM, Atmanegara B, Gani RA, Hasan I, Lesmana LA (2009). Combination of alpha-1-acid glycoprotein and alpha-fetoprotein as an improved diagnostic tool for hepatocellular carcinoma. Clin. Chim. Acta.

[8] Ohbatake Y, Fushida S, Tsukada T, Kinoshita J, Oyama K, Hayashi H (2016). Elevated alpha1-acid glycoprotein in gastric cancer patients inhibits the anticancer effects of paclitaxel, effects restored by co-administration of erythromycin. Clin. Exp. Med..

[9] Katnik I, Gerber J, Dobryszycka W (1988). Microheterogeneity of alpha 1-acid glycoprotein in the sera of patients with cancer or inflammatory states of the ovaries. Arch. Immunol. Ther. Exp. (Warsz).

[10] Piver MS, Moyer M, Diakun K, Lele SB, Chu TM (1988). Serum alpha 1-acid glycoprotein in epithelial ovarian cancer. Gynecol. Oncol..

[11] Fernandes CL, Ligabue-Braun R, Verli H (2015). Structural glycobiology of human alpha1-acid glycoprotein and its implications for pharmacokinetics and inflammation. Glycobiology.

[12] Nakano M, Kakehi K, Tsai MH, Lee YC (2004). Detailed structural features of glycan chains derived from alpha1-acid glycoproteins of several different animals: the presence of hypersialylated, O-acetylated sialic acids but not disialyl residues. Glycobiology.

[13] Zhang D, Huang J, Luo D, Feng X, Liu Y, Liu Y (2017). Glycosylation change of alpha-1-acid glycoprotein as a serum biomarker for hepatocellular carcinoma and cirrhosis. Biomark. Med..

[14] Hashimoto S, Asao T, Takahashi J, Yagihashi Y, Nishimura T, Saniabadi AR (2004). alpha1-acid glycoprotein fucosylation as a marker of carcinoma progression and prognosis. Cancer.

[15] Balmana M, Gimenez E, Puerta A, Llop E, Figueras J, Fort E (2016). Increased alpha1-3 fucosylation of alpha-1-acid glycoprotein (AGP) in pancreatic cancer. J. Proteomics.

[16] Yazawa S, Takahashi R, Yokobori T, Sano R, Mogi A, Saniabadi AR (2016). Fucosylated glycans in alpha1-acid glycoprotein for monitoring treatment outcomes and prognosis of cancer patients. PLoS One.

[17] Organization WH (2019). Breast cancer.

[18] Youlden DR, Cramb SM, Yip CH, Baade PD (2014). Incidence and mortality of female breast cancer in the Asia-Pacific region. Cancer Biol. Med..

[19] Harris L, Fritsche H, Mennel R, Norton L, Ravdin P, Taube S (2007). American society of clinical oncology 2007 update of recommendations for the use of tumor markers in breast cancer. J. Clin. Oncol..

[20] Saldova R, Asadi Shehni A, Haakensen VD, Steinfeld I, Hilliard M, Kifer I (2014). Association of N-glycosylation with breast carcinoma and systemic features using high-resolution quantitative UPLC. J. Proteome Res..

[21] Chan J, Yu D (1991). One-step isolation of alpha 1-acid glycoprotein. Protein Exp. Purif..

[22] Kim HJ, Lim SJ, Kwag HL, Kim HJ (2012). The choice of resin-bound ligand affects the structure and immunogenicity of column- purified human papillomavirus type 16 virus-like particles. PLoS One.

[23] Park MH, You JW, Kim HJ, Kim HJ (2019). IgG and IgM responses to human papillomavirus L1 virus-like particle as a function of dosing schedule and vaccine formulation. J. Microbiol..

[24] Giuliano AE, Connolly JL, Edge SB, Mittendorf EA, Rugo HS, Solin LJ (2017). Breast cancer-major changes in the American Joint Committee on Cancer eighth edition cancer staging manual. CA. Cancer J. Clin..

[25] Durham M, Regnier FE (2006). Targeted glycoproteomics: serial lectin affinity chromatography in the selection of O-glycosylation sites on proteins from the human blood proteome. J. Chromatogr. A..

[26] Choi JW, Moon BI, Lee JW, Kim HJ, Jin Y, Kim HJ (2018). Use of CA153 for screening breast cancer: An antibodylectin sandwich assay for detecting glycosylation of CA153 in sera. Oncol. Rep..

[27] Stowell SR, Ju T, Cummings RD (2015). Protein glycosylation in cancer. Annu. Rev. Pathol..

[28] Hulsmeier AJ, Paesold-Burda P, Hennet T (2007). N-glycosylation site occupancy in serum glycoproteins using multiple reaction monitoring liquid chromatography-mass spectrometry. Mol. Cell Proteomics.

[29] Lee CS, Taib NA, Ashrafzadeh A, Fadzli F, Harun F, Rahmat K (2016). Unmasking heavily O-Glycosylated serum proteins using perchloric acid: identification of serum proteoglycan 4 and protease C1 inhibitor as molecular indicators for screening of breast cancer. PLoS One.

[30] Kremer JM, Wilting J, Janssen LH (1988). Drug binding to human alpha-1-acid glycoprotein in health and disease. Pharmacol. Rev..

[31] McCurdy TR, Bhakta V, Eltringham-Smith LJ, Gataiance S, Fox-Robichaud AE, Sheffield WP (2011). Comparison of methods for the purification of alpha-1 acid glycoprotein from human plasma. J. Biomed. Biotechnol..

[32] Mackiewicz A, Mackiewicz K (1995). Glycoforms of serum alpha 1-acid glycoprotein as markers of inflammation and cancer. Glycoconj. J..

[33] Pos O, Oostendorp RA, van der Stelt ME, Scheper RJ, Van Dijk W (1990). Con A-nonreactive human alpha 1-acid glycoprotein (AGP) is more effective in modulation of lymphocyte proliferation than Con A-reactive AGP serum variants. Inflammation.

[34] Shiyan SD, Bovin NV (1997). Carbohydrate composition and immunomodulatory activity of different glycoforms of alpha1-acid glycoprotein. Glycoconj. J..

[35] Bories PN, Guenounou M, Feger J, Kodari E, Agneray J, Durand G (1987). Human alpha 1-acid glycoprotein-exposed macrophages release interleukin 1 inhibitory activity. Biochem. Biophys. Res. Commun..

